# Strontium-incorporated bioceramic scaffolds for enhanced osteoporosis bone regeneration

**DOI:** 10.1038/s41413-022-00224-x

**Published:** 2022-08-23

**Authors:** Qianju Wu, Longwei Hu, Ran Yan, Junfeng Shi, Hao Gu, Yuwei Deng, Ruixue Jiang, Jin Wen, Xinquan Jiang

**Affiliations:** 1 Department of Prosthodontics, Shanghai Ninth People’s Hospital, Shanghai Jiao Tong University School of Medicine; College of Stomatology, Shanghai Jiao Tong University; National Center for Stomatology; National Clinical Research Center for Oral Diseases; Shanghai Key Laboratory of Stomatology, Shanghai Engineering Research Center of Advanced Dental Technology and Materials, Shanghai, China; 2 Stomatological Hospital of Xiamen Medical College, Xiamen, Fujian 361008 China; 3 Department of Oral and Maxillofacial-Head and Neck Oncology, Shanghai Ninth People’s Hospital, Shanghai Jiao Tong University School of Medicine; College of Stomatology, Shanghai Jiao Tong University; National Center for Stomatology; National Clinical Research Center for Oral Diseases, Shanghai Key Laboratory of Stomatology, Shanghai Engineering Research Center of Advanced Dental Technology and Materials, Shanghai, China

**Keywords:** Bone, Bone quality and biomechanics

## Abstract

The restoration of bone defects caused by osteoporosis remains a challenge for surgeons. Strontium ranelate has been applied in preventative treatment approaches due to the biological functions of the trace element strontium (Sr). In this study, we aimed to fabricate bioactive scaffolds through Sr incorporation based on our previously developed modified amino-functional mesoporous bioactive glass (MBG) and to systematically investigate the bioactivity of the resulting scaffold in vitro and in vivo in an osteoporotic rat model. The results suggested that Sr-incorporated amino-functional MBG scaffolds possessed favorable biocompatibility. Moreover, with the incorporation of Sr, osteogenic and angiogenic capacities were upregulated in vitro. The in vivo results showed that the Sr-incorporated amino-functional MBG scaffolds achieved better bone regeneration and vessel formation. Furthermore, bioinformatics analysis indicated that the Sr-incorporated amino-functional MBG scaffolds could reduce reactive oxygen species levels in bone marrow mesenchymal stem cells in the osteoporotic model by activating the cAMP/PKA signaling pathway, thus playing an anti-osteoporosis role while promoting osteogenesis. This study demonstrated the feasibility of incorporating trace elements into scaffolds and provided new insights into biomaterial design for facilitating bone regeneration in the treatment of osteoporosis.

## Introduction

Bone defects caused by trauma, surgical tumor resection, and infectious diseases pose a significant challenge for orthopedic surgeons. Clinically, autologous bone grafts and allogeneic/allogeneic bone grafts are employed to treat bone defects^1^. However, these treatments are plagued by problems such as limited donor bone volume, postoperative complications of autologous bone grafts, and the relatively significant incidence of the immune rejection of allogeneic/allogeneic grafts. As a result, bone tissue engineering has emerged as a promising alternative to traditional bone defect treatments that can overcome these shortcomings. Various techniques, such as 3D printing and laser sintering, have been developed for fabricating composite scaffolds^2–5^. The primary tissue engineering scaffolds for bone defects are made of bioactive ceramics^6^, which are inorganic solids with crystal structures obtained through the sintering of nonmetallic salts. The human body can partially or wholly absorb bioactive ceramic scaffolds, giving such scaffolds an advantage over metal scaffolds. Additionally, the specific structure of the surface of a scaffold can be designed to have an active interfacial reaction with bone tissue. Mesoporous bioactive glass (MBG) scaffolds, possessing good biocompatibility and bone conductivity, are relatively mature in clinical application^7^. However, in the case of bone metabolic diseases, such as osteoporosis, MBG provides insufficient bone induction capability, resulting in far from desirable bone regeneration. Hence, biomimetic strategies have been used to construct multifunctional active biomaterials for oral and maxillofacial bone restoration^8^. At present, such strategies include surface morphology alteration and chemical component regulation. Surface morphology alteration typically involves micro/nanostructure modification or functional molecular coatings. Unfortunately, the biological performance of biomaterials subjected to surface morphology alteration remains insufficient, particularly in vivo. Chemical component regulation typically involves the application of various growth factors and polymer materials. However, the complicated preparation methods, high cost, insufficient local concentrations, and unexpected side effects restrict the clinical application of chemical component regulation.

In our previous research, we transformed MBG into N-MBG by grafting an amino group onto MBG with a simple powder pressing process, and the performance of N-MBG was systematically evaluated. Our results showed that N-MBG exhibited osteogenic capability to some extent when loaded with an anti-osteoporosis drug^9^. However, it is still challenging to regenerate a sufficient amount of bone in the osteoporotic oral and maxillofacial regions. Strontium ranelate has been traditionally utilized to treat osteoporosis because it releases Sr ions, which directly prevent osteoclast activation and promote osteoblast differentiation^10–13^. Hence, it is reasonable to speculate that N-MBG loaded with Sr ions (Sr-N-MBG) would be promising for bone regeneration in osteoporosis. Therefore, the present study aimed to fabricate novel scaffolds incorporating Sr and systematically investigate the biological performance of the scaffolds in an osteoporosis model.

Although there is some evidence that the incorporation of Sr into scaffolds could be a promising approach for bone regeneration in the laboratory, most of the studies providing this evidence were phenomenological. Little research has focused on gene expression changes or signaling pathway variations. Moreover, the lack of bioinformatics analysis has led to an incomprehensive understanding of the interactions between biomaterials and surrounding cells. To address these gaps in the corresponding knowledge, we aimed to elucidate the gene expression and signaling pathway changes of bone marrow mesenchymal stem cells (BMSCs) from an osteoporotic model induced by ovariectomy (OVX), hereafter referred to as OVX BMSCs, cultured on our novel scaffolds, as this could provide new insights into biomaterial design for improved osteogenesis.

## Results

### Biocompatibility of fabricated scaffolds

**Fig. 1:**
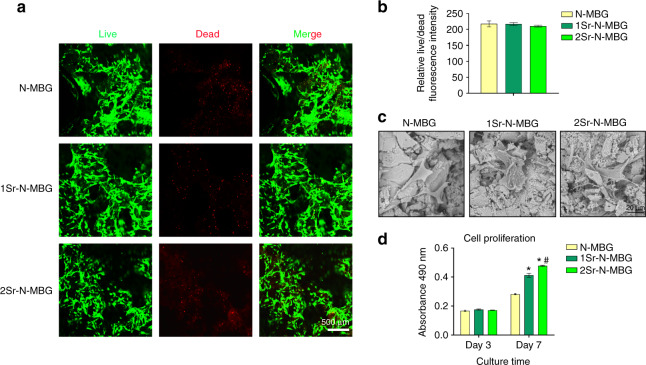
Biocompatibility evaluation of fabricated scaffolds. **a** Live/dead assay of osteoporotic bone marrow mesenchymal stem cells (OVX BMSCs) seeded on each sample. **b** Corresponding quantitative analysis of live/dead staining. **c** Cellular morphology on different scaffolds by SEM. **d** Results of the CCK-8 assay for the proliferation of OVX BMSCs. Notes: The symbol ⋆ indicates *P* < 0.05 compared with the N-MBG group, and the symbol # indicates *P* < 0.05 compared with the 1Sr-N-MBG group

The biocompatibility of the scaffolds was first investigated. As shown in Fig. 1a and b, live cells displayed green fluorescence, and dead cells were stained red. There was no significant difference between live and dead cells, confirming the lack of cytological toxicity of the fabricated scaffolds. The attachment and morphology of cells on N-MBG, 1Sr-N-MBG, and 2Sr-N-MBG scaffolds were observed by scanning electron microscopy (SEM) (Fig. 1c). After culturing for three days, OVX BMSCs were attached to each type of scaffold surface and demonstrated a well-spread morphology, as indicated by the stretching of pseudopodia. The proliferation activities determined by Cell Counting Kit-8 (CCK-8) assays for the cells on Days 3 and 7 are shown in Fig. 1c. Each type of scaffold could support cell proliferation. Remarkably, the proliferation rates for Sr-N-MBG scaffolds (1Sr-N-MBG and 2Sr-N-MBG) were markedly higher than those for the N-MBG group at Day 7 (*P* < 0.05), and the 2Sr-N-MBG group showed the most enhanced proliferation capability.

### Evaluating the osteogenic capability of the prepared materials in vitro

**Fig. 2:**
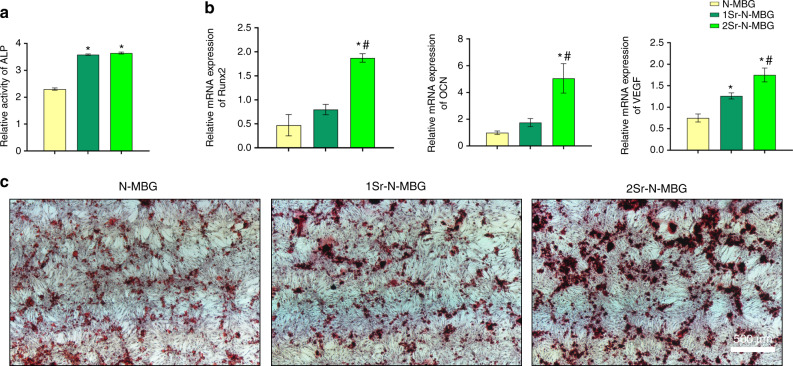
Osteogenic capability evaluation. **a** Relative ALP activity of cells cultured on each scaffold. **b** Gene expression of the osteogenesis-related markers Runx2 and OCN and the angiogenic marker VEGF. **c** ARS investigation of calcium deposition. Notes: The symbol ⋆ indicates *P* < 0.05 compared with the N-MBG group, and the symbol # indicates *P* < 0.05 com*p*ared with the 1Sr-N-MBG group

The alkaline phosphatase (ALP) activity of OVX BMSCs cultured on scaffolds for seven days was measured (Fig. 2a), and the Sr-N-MBG scaffolds demonstrated significantly higher ALP activity (*P* < 0.05). Moreover, cell differentiation was further detected by determining the expression of osteogenic markers on Day 7 (Fig. 2b). The osteogenesis-related genes runt-related transcription factor 2 (Runx2) and osteocalcin (OCN)^14^ were upregulated on 2Sr-N-MBG scaffolds with a significant difference (*P* < 0.05). They were also upregulated on 1Sr-N-MBG scaffolds, although there was no significant difference between the 1Sr-N-MBG and N-MBG groups. Additionally, vascular endothelial growth factor (VEGF), one of the most important markers for angiogenesis^15^, showed an increased expression pattern in a dose-dependent manner with regard to Sr incorporation. To evaluate the influence of Sr ions released from scaffolds, OVX BMSCs were incubated in the extract of each scaffold material, and their calcium deposition ability was measured by Alizarin red S (ARS) staining. As presented in Fig. 2c, the calcium deposition-positive area of OVX BMSCs cultured in the extract from Sr-N-MBG scaffolds was higher, indicating that the incorporation of Sr into scaffolds could facilitate the osteogenic differentiation of stem cells.

### Determination of the angiogenic ability of the prepared materials extracted in vitro

**Fig. 3:**
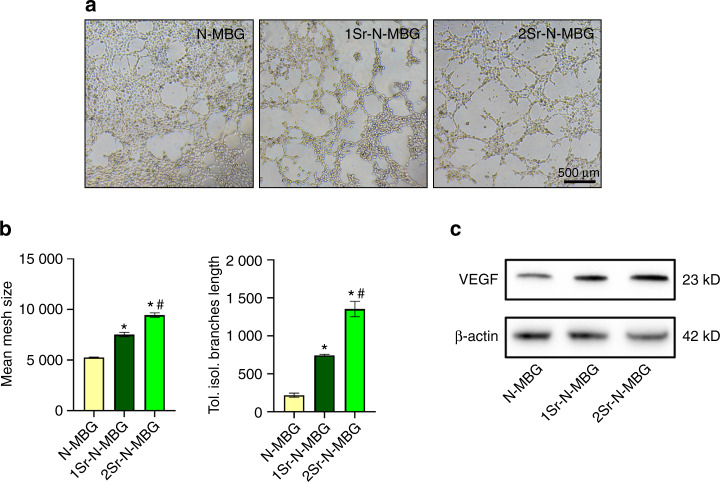
The extract from Sr-N-MBG scaffolds stimulated the angiogenic ability of HUVECs in vitro. **a** Tube formation assay of HUVECs cultured in different scaffold extracts for 4 h. **b** Quantitative analysis of the mean size of meshes and total length of isolation branches. Notes: *P < 0.05 versus the control N-MBG group; #*P* < 0.05 versus the 1Sr-N-MBG group. **c** Western blot analysis for the expression of VEGF

Figure 3a shows the meshed networks of human umbilical vein endothelial cells (HUVECs) formed on Matrigel. The HUVECs cocultured with 1Sr-N-MBG extract formed larger meshes and more isolation branches. In contrast, the HUVECs cocultured with 2Sr-N-MBG extract showed the most remarkable results by quantitative analysis (Fig. 3b). Moreover, the HUVECs cocultured with both Sr-N-MBG scaffold extracts displayed an upregulated tendency of vascular endothelial growth Factor (VEGF) expression (Fig. 3c).

### Comparative ectopic osteogenesis

**Fig. 4:**
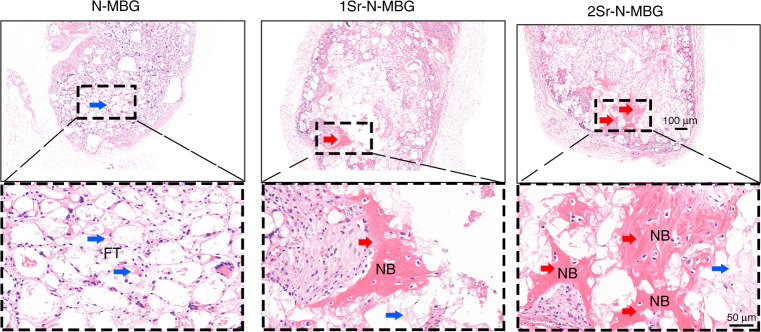
Representative images for ectopic osteogenesis capability evaluation for different groups and corresponding higher magnification sections in the lower row. FT: fibrous tissue (blue arrows); NB: new bone (red arrows)

Each OVX BMSC-seeded scaffold was implanted subcutaneously into nude mice for four weeks, and samples were then extracted and used to detect ectopic bone formation. The hematoxylin-eosin (HE) staining results showed that new bone formation was detectable in Sr-N-MBG scaffolds, and more mature bone tissue could be observed with increasing Sr incorporation. In contrast, only fibrous connective tissue could be found in the N-MBG group (Fig. 4).

### Bone tissue regeneration for critical-sized calvarial defects

#### Microcomputed tomography (micro-CT) measurement

**Fig. 5:**
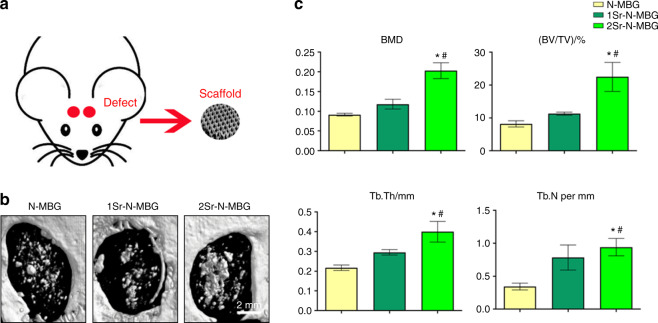
Bone tissue regeneration for critically sized calvarial defects. **a** Schematic diagram. **b** Typical photographs of defects restored by the N-MBG, 1Sr-N-MBG, and 2Sr-N-MBG treatments. **c** Corresponding calculated quantitative variables of bone mineral density (BMD), bone volume to total volume (BV/TV) ratio, trabecular thickness (Tb. Th), and trabecular number (Tb. N). Notes: The symbol ⋆ indicates *P* < 0.05 compared with the N-MBG group, and the symbol # indicates *P* < 0.05 com*p*ared with the 1Sr-N-MBG group

The newly formed bone of the defect sites was scanned. Then, the morphology was reconstructed by micro-CT, and the newly formed bone was analyzed via morphometrical analysis. After 8 weeks, the newly formed bone area in the Sr-N-MBG scaffolds was more significant than that in the N-MBG scaffolds (Fig. 5b). The corresponding quantity parameters showed similar results, indicating that the Sr-N-MBG scaffolds improved new bone formation. This tendency turned out to be Sr dose-dependent, as the 2Sr-N-MBG group exhibited a significantly greater bone mineral density (BMD), bone volume to total volume (BV/TV) ratio, trabecular thickness (Tb. Th), and trabecular number (Tb. N) compared to the other two groups, and 1Sr-N-MBG exhibited the upregulation of these parameters but with no significant difference compared to the N-MBG group (Fig. 5c).

#### Histological analysis

**Fig. 6:**
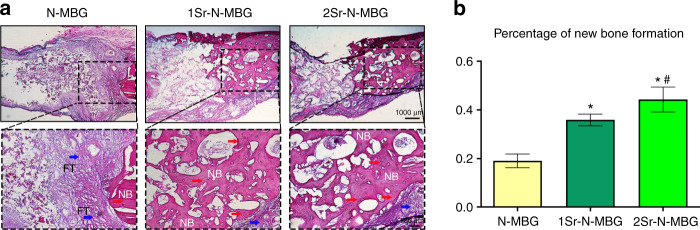
Histological analysis of bone tissue regeneration for critically sized calvarial defects. **a** Typical photographs by HE analysis for each group. **b** Corresponding quantitative variables. FT: fibrous tissue (blue arrows); NB: new bone (red arrows). Notes: The symbol ⋆ indicates *P* < 0.05 compared with the N-MBG group, and the symbol # indicates *P* < 0.05 compared with the 1Sr-N-MBG group

**Fig. 7:**
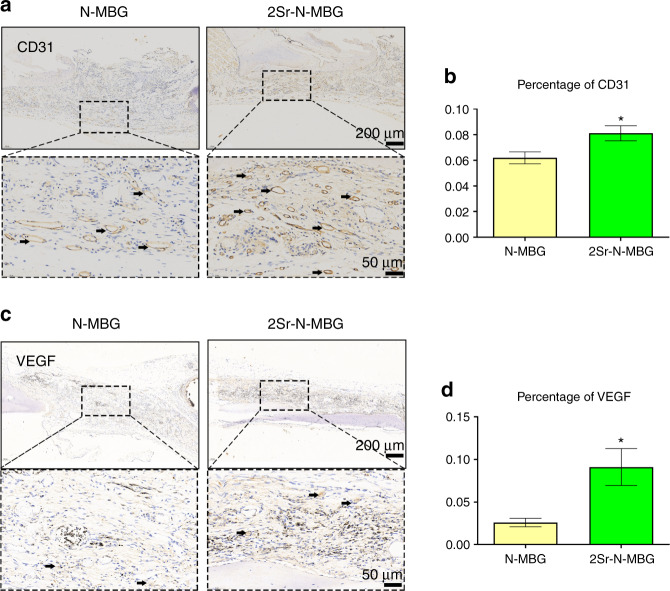
Angiogenic activity by IHC determination. **a**, **b** Expression of CD31 and the corresponding quantitative results. **c**, **d** Expression of VEGF and the corresponding quantitative results. Notes: The symbol ⋆ indicates *P* < 0.05 compared with the N-MBG group

The histological observation of specimens stained with HE clearly illustrated that a much greater amount of newly formed bone was observed in the Sr-N-MBG scaffolds. In contrast, a small amount of bone combined with fibrous tissue was found in the N-MBG group, and the most superior connectivity of new bone was observed in the 2Sr-N-MBG group (Fig. 6). More blood vessel structures (indicated by arrows) could be detected in the 2Sr-N-MBG group. The higher intensities of angiogenic markers CD31 and VEGF also implied that new blood vessel formation was active in the 2Sr-N-MBG group, suggesting the promise of locally applying Sr to promote angiogenesis (Fig. 7).

### Underlying mechanism exploration

**Fig. 8:**
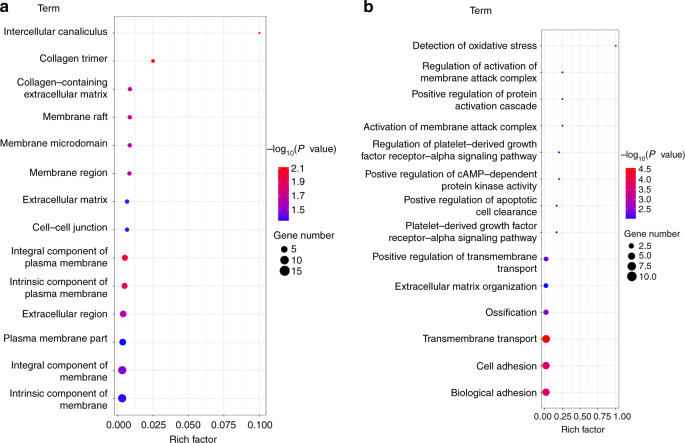
RNA-seq analysis between the N-MBG and 2Sr-N-MBG groups for comparison. **a** Statistics of GO enrichment of CC. **b** GO enrichment analysis of BP

To determine the underlying mechanism of enhanced bone regeneration for the Sr-N-MBG scaffolds, RNA-seq was performed on the N-MBG and 2Sr-N-MBG groups for comparison. A total of 127 genes were recognized as differentially expressed (59 up and 68 down) (Fig. S2a). Gene ontology (GO) enrichment analysis was carried out to determine the functional changes underlying these genomic data, and a list of GO terms was generated accordingly. The GO enrichment analysis indicated a marked difference in the transcriptional biological function of these two groups, particularly for cellular component (CC) activity (Fig. S2b). In the GO enrichment evaluation of CC, it was observed that CCs including intercellular canaliculus, collagen trimer, and collagen-containing extracellular matrix trimer varied significantly in the 2Sr-N-MBG group, which could facilitate subsequent cellular differentiation for osteogenic activity (Fig. 8a). Moreover, according to the GO enrichment evaluation of biological process (BP), the category of detecting oxidative stress showed a significant difference between the N-MBG group and the Sr-N-MBG scaffold, implying that the Sr-N-MBG scaffold could modulate BP (Fig. 8b).

**Fig. 9:**
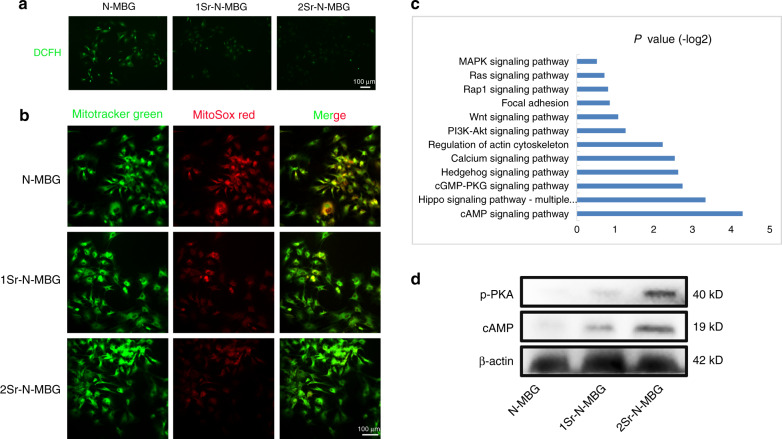
Sr-N-MBG scaffolds reduced ROS levels by activating the cAMP/PKA signaling pathway in OVX BMSCs. **a** Intracellular ROS detection. **b** Mitochondrial ROS detection. **c** KEGG pathway determination**. d** Western blot analysis for investigating the expression level of cAMP and PKA phosphorylation

Subsequently, the intracellular reactive oxygen species (ROS) in OVX BMSCs were visually labeled with the fluorescent probe 2′,7′-dichlorofluorescein diacetate (DCFH-DA) to identify ROS-scavenging properties (Fig. 9a). The Sr-N-MBG scaffolds exhibited lower fluorescence intensity with Sr incorporation. Since mitochondria are the primary source of ROS generation, a highly selective fluorescent dye (MitoSox Red) was utilized to investigate mitochondrial ROS levels. As predicted, the Sr-N-MBG scaffolds displayed a remarkably better reduction in mitochondrial ROS accumulation than the N-MBG group (Fig. 9b). The acquired results demonstrated that the Sr-N-MBG scaffolds resisted oxidative stress by scavenging ROS. Furthermore, Kyoto Encyclopedia of Genes and Genomes (KEGG) pathway analysis indicated that the genes associated with cAMP pathways showed significantly different expression patterns between the N-MBG group and the Sr-N-MBG scaffold (Fig. 9c). Western blotting was subsequently conducted to demonstrate the results of KEGG. The cAMP and PKA phosphorylation levels were highly expressed in the Sr-N-MBG scaffolds, which could be a potential underlying mechanism for the enhancement of osteogenesis against osteoporosis (Fig. 9d).

## Discussion

Osteoporosis is a significant disease that widely affects middle-aged and older patients. Osteoporosis reduces bone mass, increases bone brittleness, and particularly weakens the regeneration ability of damaged bone. Hence, it has high morbidity, mortality, and disability rates^16,17^. The restoration of bone tissue in osteoporosis-affected patients remains a significant problem. Strontium ranelate is a therapeutic agent that can reduce the risk of fractures for osteoporosis patients because of the biological roles of Sr ions in bone tissue metabolism^18^, specifically their promotion of the proliferation of osteoblasts and simultaneous inhibition of the activity of osteoclasts^13,19^. Nevertheless, this systemic medication not only affects the dynamic balance of bone metabolism but also yields some unexpected side effects, such as cardiac failure^20,21^. Thus, there is a need to maintain a local release pattern of Sr, which could be achieved by incorporating Sr into biomaterials^22,23^. In our previous work, we fabricated N-MBG as a model scaffold and demonstrated that it exhibited osteogenic capability to some extent, and it had a satisfactory loading capacity for drugs or growth factors. In the present study, we incorporated Sr into the N-MBG scaffold and evaluated the feasibility of restoring calvarial defects in osteoporosis models. Our objective was to provide new guidelines for designing bioactive scaffolds for restoring other parts of bone defects in the human body.

The porous scaffolds fabricated in this study had a rough, irregular surface. Interestingly, with the incorporation of Sr ions, a unique homogeneous nanoneedle morphology emerged. Regarding ion incorporation, cytological toxicity must be taken into consideration carefully. A series of past studies investigated the feasibility of Sr incorporation. For example, Pontremoli concluded that final release of Sr at concentrations of 1.6–4.4 μg·mL^−1^ had the potential to stimulate osteogenic response without causing side effects^24^. Zhao showed in a recent study that a 0.5–1.5 μg·mL^−1^ concentration released from a Sr-bioceramic could be a safe bone substitute and promote local bone regeneration^25^. The modified scaffolds in the present study displayed a sustained release pattern of Sr, and the concentration result was approximately in agreement with those of with previous reports, making it reasonable to speculate that the ions released could have cellular activity without imposing cytological toxicity (Fig. S1)^26–28^. It is well known that the proliferation and differentiation of bone-forming cells have significant roles in the early phase of bone mineralization, and these biological processes are manipulated by cell interactions with the underlying matrix^29,30^. Hence, the proliferation and potential osteogenic capability of OVX BMSCs cultured on Sr-N-MBG scaffolds were further investigated in the present study. In vitro experiments showed that the incorporation of Sr did not hamper cell viability but promoted proliferation. Moreover, the incorporation of Sr was favorable for cell spreading and induced extraordinary stretching morphology, suggesting the excellent biocompatibility of the Sr-N-MBG scaffolds. Furthermore, the ALP activity and improved osteogenesis-related marker expression of the OVX BMSCs revealed the superior osteogenic ability of the Sr-N-MBG scaffolds. Additionally, the OVX BMSCs incubated in the Sr-N-MBG scaffold extracts showed a higher calcium deposition ability than those incubated in the N-MBG scaffold extracts, emphasizing the positive influence of Sr. Because of the crosstalk between osteogenesis and angiogenesis, the modification of scaffold materials for promoting angiogenic capability in bone defect regions is favorable for bone regeneration^31,32^. In the present study, real-time PCR showed that VEGF expression was significantly upregulated in the OVX BMSCs cultured on Sr-N-MBG scaffolds. Moreover, the angiogenic effects of the scaffold material extract on HUVECs were subsequently detected in vitro via a tube formation assay. The results revealed that the Sr-N-MBG extract could effectively promote the formation of tube-like structures by HUVECs on the Matrigel matrix. Furthermore, the expression level of VEGF protein, acting as the crucial angiogenic regulator that enhances endothelial cell proliferation and initiates angiogenesis, displayed an upregulated tendency in a Sr dose-dependent manner, indicating the angiogenesis effect of the Sr-N-MBG scaffold material and laying a foundation for bone regeneration^33^.

Ectopic osteogenesis was analyzed to investigate the potential clinical applications of the Sr-N-MBG scaffolds for osteoporosis patients. The results indicated that the Sr-N-MBG scaffolds could facilitate ectopic bone formation. Moreover, critically sized calvarial defects, which were subsequently repaired by biomaterials, were also constructed in an animal model. After eight weeks of implantation, micro-CT and histological examination vividly demonstrated that newly formed bone area and quality were higher around the Sr-N-MBG scaffolds than around the N-MBG scaffolds, as indicated by a series of parameters, such as BMD, BV/TV, Tb. Th, and Tb. N. Excitingly, more intensive blood vessel formation occurred in bone defect areas when Sr-N-MBG scaffolds were implanted. Generally, osteogenesis and angiogenesis have an intimate relationship. Their biological interaction is manipulated by a precise regulating network of growth factors, such as VEGF^34,35^. Previous research has demonstrated that strontium ranelate can activate the PI3K/AKT/mTOR signaling pathway and potentially facilitate angiogenic activity^33^. Some studies have shown that strontium ions released by the degradation of materials could significantly increase the polarization trends of macrophages from the M1-type to the M2-type and promote the expression of anti-inflammatory cytokines, which could establish a suitable local microenvironment favoring blood vessel formation and maturation^36^. Even though such studies have demonstrated that Sr could stimulate angiogenesis in healthy models, less attention has been given to determine whether this ability could still be achieved in osteoporotic models. The results of the present study regarding angiogenesis and new bone formation suggest that the stimulatory effect of Sr on vascularization could also be obtained in vivo, as we expected.

The biological reactions between host cells and the surrounding microenvironment are complicated and remain a popular research topic. To investigate the mechanisms by which Sr-N-MBG scaffolds improve bone defect repair for the treatment of osteoporosis, RNA-seq genome-wide analysis was conducted^37^. It is worth noting that the genes associated with CCs significantly differed between the N-MBG and 2Sr-N-MBG scaffold groups, suggesting cellular conformational changes. Scientists have demonstrated that mechanotransduction is the underlying mechanism by which cells perceive cues from extracellular environments and transform them into intracellular signals to initiate biological responses. During the mechanotransduction process, the cytoskeleton structure plays a significant role and has been demonstrated to act as a mechanotransmitter by conformational alterations. Previous studies have suggested that increased cytoskeletal activity is closely linked with osteogenesis for human MSCs^38^. Moreover, recent reports have implied that osteogenic activity, which is associated with cytoskeletal alterations, is triggered by the intracellular β-catenin signaling pathway^39^. Based on our experimental data, we speculated that the needlelike-structured topography of the Sr-N-MBG scaffolds played a significant role in promoting the cytoskeleton activity of cells at an early stage. Then, Sr was released from the material surface, playing an essential role in osteogenesis. Therefore, it was reasonable to attribute the better osteogenic activity of Sr-N-MBG scaffolds to the synergistic effect of the topography and the dissolution products of Sr^18,40^.

ROS are essential products of cellular redox processes during biological metabolism. Intracellular glutathione, peroxidase, catalase, peroxidase redox protein, and antioxidants are scavengers of ROS, meaning they convert ROS into oxygen and water to maintain the redox homeostasis of the microenvironment^41,42^. When excessive ROS accumulate and cannot be eliminated, oxidative stress occurs. Studies have shown that osteoporotic patients exhibit higher levels of oxidative stress in vivo. Under oxidative stress conditions, antioxidants in osteoporotic cells cannot eliminate excessive ROS, which can induce the apoptosis of osteoblasts and osteocytes while promoting the formation of osteoclasts and enhancing their activities. As a result, reducing excessive ROS levels to mitigate oxidative stress serves as a promising strategy for the treatment of osteoporosis^43^. In the present study, bioinformatics analysis suggested that the Sr-N-MBG scaffolds might regulate biological oxidative stress. Hence, the intracellular and mitochondrial ROS levels were measured, and the results showed that Sr-N-MBG scaffolds effectively reduced the ROS levels in OVX BMSCs. According to the KEGG results, there was a significant difference in the expression of genes associated with the cAMP signaling pathway. The first identified second messenger, cAMP, plays an essential role in intracellular biological responses to various extracellular stimuli and regulates multiple cellular processes. Studies have shown that cAMP can regulate oxidative stress responses and eliminate ROS by activating downstream PKA, thus reducing oxidative stress in osteoporotic cells^44,45^. Western blot analysis confirmed that the Sr-N-MBG scaffolds could activate cAMP and PKA phosphorylation in OVX BMSCs, consistent with the bioinformatics results. In conclusion, the Sr-N-MBG scaffolds prepared in this study could reduce ROS levels by activating the cAMP/PKA signaling pathway, thereby playing an anti-osteoporosis role while promoting osteogenesis. Therefore, our study provides a basis for the future development of antioxidative stress biomaterials for facilitating bone regeneration in the treatment of osteoporosis^46–48^.

## Materials and methods

### Fabrication and characterization of materials

The primary scaffolds were synthesized by a straightforward one-step approach as previously described^49^. Briefly, 8.0 g of P123 was placed into a mixture of HNO_3_ (120 mL, 2 mol·L^−1^) and distilled water (30 mL, 2 mol·L^−1^) and stirred until dissolved. Then, SrCl·6H_2_O (0 g, 0.67 g, or 1.34 g to form three distinct mixtures), 13.4 tetraethoxysilane, 2.8 g Ca(NO_3_)·4H_2_O, and 1.46 g triethyl phosphate were added while stirring at room temperature. The three mixtures were transferred into an autoclave and heated at 700 °C for 5 h. Next, scaffolds were fabricated by a simple powder pressing process, and polyvinylpyrrolidone and polyethylene glycol were used as adhesives and pore-forming agents. Subsequently, 3-aminopropyltrimethoxysilane (97%) was employed to graft the amino-functional groups onto the fabricated scaffolds. The three groups of fabricated scaffold samples made from the three aforementioned mixtures were denoted as N-MBG, 1Sr-N-MBG, and 2Sr-N-MBG, respectively^50^. SEM (Hitachi S4800 electron microscope, Tokyo, Japan) was employed to investigate their surface morphology, and inductively coupled plasma–mass spectrometry (NuInstruments, Wrexham, UK) was used to detect the released concentration of Sr ions by collecting the extract liquid of different scaffolds on Days 1, 3, 5, 7, and 14.

### Preparation of osteoporotic model and culture of OVX BMSCs

Experimental protocols were approved by the Animal Care and Experiment Committee, Ninth People’s Hospital^51^, and all operations were carried out under sterile conditions. An osteoporotic model was induced by OVX in female Sprague–Dawley rats. Briefly, the enterocoel was exposed by a minimally bilateral lumbar incision, and then ovaries were pulled out and removed once vessel ligation was finished. Antibiotics were administered postoperatively. Three months after ovariectomy, the animals were sacrificed, the femora were harvested, and both ends were cut off. The bone marrow from the exposed medullary canal was flushed out and centrifuged to obtain a pellet of primary OVX BMSCs, which was suspended, placed in a dish, and incubated. The culture medium was replaced every 2–3 days to discard nonadherent cells under culture conditions (5% CO_2_ and 37 °C). Cells in passages 2–4 were used for the following experiments^52^.

### Biocompatibility investigation

OVX BMSCs were seeded onto different samples at a density of 2.0 × 10^4^. A live/dead assay was carried out to determine cellular fate by a Calcein/PI Cell Viability/Cytotoxicity Assay Kit after culturing for three days. According to the manufacturer’s instructions, the working solution was prepared and added to different samples (200 μL per well). After incubation for 30 min, Calcein AM was used to stain living cells, which showed green fluorescence, while red fluorescence indicated dead cells labeled by propidium iodide (PI). The relative live/dead fluorescence intensity was calculated, and statistical analysis was performed. The cell morphology was also observed by SEM. Each sample was washed with phosphate-buffered saline, fixed in 3% glutaraldehyde, dehydrated in increasing concentrations of ethanol, and then sputter-coated with platinum for final observations. The proliferation capability was detected by Cell Counting Kit-8 (CCK-8) analysis. On Days 3 and 7, CCK-8 (200 μL per well) was added to DMEM and incubated for 1 h. Afterward, the absorbance value was measured at 490 nm by an ELX ultra microplate reader (BioTek, USA). All operations were performed in triplicate.

### Osteogenic ability evaluation in vitro

For the alkaline phosphatase (ALP) activity quantitative assay, cells seeded on samples were collected and incubated with p-nitrophenyl phosphate (pNPP, Sigma, St. Louis, MO, USA) at 37 °C for 30 min. The results were measured and displayed as optical density (OD) values at 405 nm. The Bradford method was used to detect the total protein content. All operations were performed in triplicate. Real-time PCR assays were also carried out. Briefly, the RNA of cells was extracted after 14 days of culturing by TRIzol (Invitrogen, USA). The harvested RNA was used to synthesize DNA with a cDNA synthesis kit (Takara, Japan). By the reverse transcription-polymerase chain reaction system (Bio-Rad, USA), the expression levels of osteogenic and angiogenic markers (OCN, Runx2, and VEGF) were detected. The primer sequences are shown in Table S1. All expression levels were normalized to β-actin, and the operations were performed in triplicate. Moreover, to investigate the biological performance of the Sr ions released from the scaffolds, cells were incubated in the extracted liquid of each material for 14 days, and ARS staining was used to investigate extracellular matrix mineralization. Briefly, cells were fixed in 4% paraformaldehyde, and a 0.1% ARS working solution was prepared according to the manufacturer’s instructions and used for the following study.

### Angiogenetic capability ability evaluation in vitro

Tube formation analysis was conducted for HUVECs cultured in the extract of Sr-N-MBG scaffolds to determine their angiogenic ability in vitro. All the experimental instruments, including culture plates and tips, were precooled at −20 °C before the plates were coated with Matrigel (Corning, USA) at 150 μL per well. The plates were incubated for 30 min at 37 °C to solidify the Matrigel. Then, HUVECs (ScienCell, USA) were suspended and seeded onto the preplated Matrigel at 2 × 10^5^ cells per well. After the cells were attached, the scaffold material extracts were added to the medium, and microscopy was used to detect tube formation at 4 h. Furthermore, western blotting was carried out to detect the VEGF expression levels in the HUVECs. Briefly, HUVECs were cultured in Sr-N-MBG scaffold extract for seven days. The cells were lysed with the protein extraction reagent, which contained a protease inhibitor cocktail, phosphatase inhibitor cocktail, and phenylmethanesulfonylfluoride (PMSF) (Kangchen, China), and the concentration of acquired protein was calculated by a Bio-Rad protein assay kit. Equivalent amounts of protein were separated by SDS-polyacrylamide gel electrophoresis (PAGE) and electrotransferred to polyvinylidene difluoride (PVDF) membranes (Pall, USA). The PVDF membranes were incubated with specific primary VEGF antibodies (CST, USA) at a dilution of 1:1 000 overnight at 4 °C. Finally, the PVDF membranes were visualized by horseradish peroxidase (HRP)-conjugated secondary antibodies (Beyotime, China) by ECL Plus reagents (Amersham Pharmacia Biotech, USA) using a UVItec ALLIANCE 4.7 gel imaging system.

### Surgical procedure for ectopic osteogenesis in nude mice

Six nude mice were selected and anesthetized for implantation. In each mouse, a subcutaneous dorsal pocket was created by blunt dissection, three groups of scaffolds seeded with OVX BMSCs were implanted, and the wounds were sutured. Four weeks later, the mice were sacrificed, and the scaffolds together with 1-cm soft tissue were extracted and fixed. After decalcification, they were embedded in paraffin and subsequently fabricated into 4-μm-thick slices by a microtome (Leica, Germany). The sliced sections were subjected to HE staining to investigate bone formation.

### Animal surgical procedure and histological investigation

Critically sized defects of 5 mm in diameter were created in calvarial bones in the osteoporotic model. Subsequently, each scaffold (5 mm in diameter) was used to implant the defects, and antibiotics were administered postsurgically^53^. After eight weeks, the animals were sacrificed, and the skulls of the surgery regions were harvested. The bone regeneration outcome was detected by micro-CT (SkyScan 1176, Belgium) and 3-D Creator software (Scanco Medical, Switzerland). Moreover, as previously described, corresponding parameters (BMD, BV/TV, Tb. Th, and Tb. N) were also calculated to obtain quantitative results. The skull specimens were prepared into halves along the sagittal plane for subsequent histological observation. After decalcification, they were embedded in paraffin and subsequently trimmed into 4-μm-thick slices. The slice sections were subjected to HE staining and immunohistochemical (IHC) staining for CD31 and VEGF (Abcam) to investigate bone formation and angiogenesis. The quantitative parameters were measured using Image-Pro Plus™ (Media Cybernetics, Silver Springs, MD, USA).

### RNA-Seq analysis

OVX BMSCs were seeded onto the N-MBG and 2Sr-N-MBG scaffolds and incubated, and total RNA was isolated by TRIzol (Invitrogen, USA) according to the manufacturer’s instructions. The qualities of the RNA samples were assessed, and those with a 28S/18S RNA ratio ≥0.7 were acquired^54^. For sequence and primary analysis, Cutadapt software was employed to remove the reads that contained adapter contamination, and then HISAT2 software was used to map the reads to the genome. Then, all transcriptomes from each sample were merged to reconstruct a comprehensive transcriptome. The differentially expressed mRNAs were selected with a fold change >2 or fold change <0.5 and *P* < 0.05. GO enrichment and KEGG enrichment analyses of the differentially expressed mRNAs were carried out.

### Detection of intracellular and mitochondrial ROS and the expression of cAMP/p-PKA

The level of intracellular ROS production was determined by labeling the OVX BMSCs seeded on the N-MBG and Sr-N-MBG scaffolds with 2′,7′-dichlorodihydrofluorescein diacetate (DCFH-DA) (10 × 10^−6^ m, Thermo Scientific) for 30 min. Moreover, to investigate the levels of mitochondrial ROS, the cells were counterstained with the selective fluorescent dye MitoSOX Red (2.5 × 10^−6^ m, Thermo Scientific), which targets superoxide production in the mitochondria of living cells. MitoTracker Green was employed to label the mitochondria (150 × 10^−9^ m, Thermo Scientific) via incubation for 30 min at 37 °C. According to the KEGG enrichment analysis results, western blotting was used to detect cAMP/p-PKA expression. Briefly, OVX BMSCs were cocultured in extracts of different materials for seven days. The cells were lysed with protein extraction reagent, and the concentration of acquired protein was calculated. As mentioned above, equivalent amounts of protein were separated by SDS–PAGE and electrotransferred to PVDF membranes incubated overnight at 4 °C with specific primary antibodies against cAMP and p-PKA (CST, USA) at a dilution of 1:1 000. Finally, they were visualized by HRP-conjugated secondary antibodies (Beyotime, China) by ECL Plus reagents (Amersham Pharmacia Biotech, USA) using the UVItec ALLIANCE 4.7 gel imaging system.

### Statistical analysis

SPSS software (SPSS Inc., USA) was used, and the statistical significance was determined by analysis of variance and *t* tests. The symbol ⋆ indicates *P* < 0.05 compared with the N-MBG group, and the symbol # indicates *P* < 0.05 compared with the 1Sr-N-MBG group.

## Supplementary information

Supplemental Material
