# Supplementary material for: Strontium-incorporated bioceramic scaffolds for enhanced osteoporosis bone regeneration
Source: Bone Res. 2022 Aug 23;10:55. doi: 10.1038/s41413-022-00224-x (PMC9399250; doi:10.1038/s41413-022-00224-x)
Supplement: Supplementary file 1 — Supplemental Material [file 41413_2022_224_MOESM1_ESM.doc]

**Strontium-incorporated bioceramic scaffolds in enhanced osteoporosis bone regeneration**

Qianju Wu^1,2^, Longwei Hu^3^, Ran Yan^1^, Junfeng Shi^1^, Hao Gu^1^,

Yuwei Deng^1^, Ruixue Jiang^1^, **Jin Wen^1*^, Xinquan Jiang^1*^**

1, Department of Prosthodontics, Shanghai Ninth People's Hospital, Shanghai Jiao Tong University School of Medicine; College of Stomatology, Shanghai Jiao Tong University; National Center for Stomatology; National Clinical Research Center for Oral Diseases; Shanghai Key Laboratory of Stomatology; Shanghai Engineering Research Center of Advanced Dental Technology and Materials, Shanghai, P.R. China.

2, Stomatological Hospital of Xiamen Medical College. Xiamen, Fujian Province 361008, China.

3, Department of Oral and Maxillofacial-Head and Neck Oncology, Shanghai Ninth People's Hospital, Shanghai Jiao Tong University School of Medicine; College of Stomatology, Shanghai Jiao Tong University; National Center for Stomatology; National Clinical Research Center for Oral Diseases; Shanghai Key Laboratory of Stomatology; Shanghai Engineering Research Center of Advanced Dental Technology and Materials, Shanghai, P.R. China.

These authors contributed equally: Qianju Wu^1,2^ Longwei Hu^3^

**Corresponding Authors:**

**Prof. Xinquan Jiang**, Department of Prosthodontics, Ninth People’s Hospital affiliated to Shanghai Jiao Tong University, School of Medicine, 639 Zhizaoju Road, Shanghai 200011, China. E-mail: [xinquanjiang@aliyun.com](mailto:xinquanjiang@aliyun.com). Tel: +86 21 63135412. Fax: +86 21 63136856.

**Dr. Jin Wen**, Department of Prosthodontics, Ninth People’s Hospital affiliated to Shanghai Jiao Tong University, School of Medicine, 639 Zhizaoju Road, Shanghai 200011, China. E-mail: echomet@126.com. Tel: +86 21 63135412. Fax: +86 21 63136856.

**Table 1.** Primers for real-time polymerase chain reaction (PCR).

| **Gene** | **Prime sequence**  **(F, forward; R, reverse)** | **Product size (bp)** | **Accession number** |
| --- | --- | --- | --- |
| **β-Actin** | F: AGGGAGTGATGGTTGGAATG  R: GATGATGCCGTGTTCTATCG | 107 | NM_031004.2 |
| **RUNX-2**  **OCN** | F: CCGAGACCAACCGAGTCATT  R: CACTGCACTGAAGAGGCTGT  F: AGTAAGGTGGTGAATAGACTCCG  R: GGTGCCATAGATGCGCTTG | 114  172 | NM_001278483.1  NM_013414.1 |
| **VEGF** | F: TTGAGTTGGGAGGAGGATGT  R: TGGCAGGCAAACAGACTTC | 115 | NM_001110333.1 |


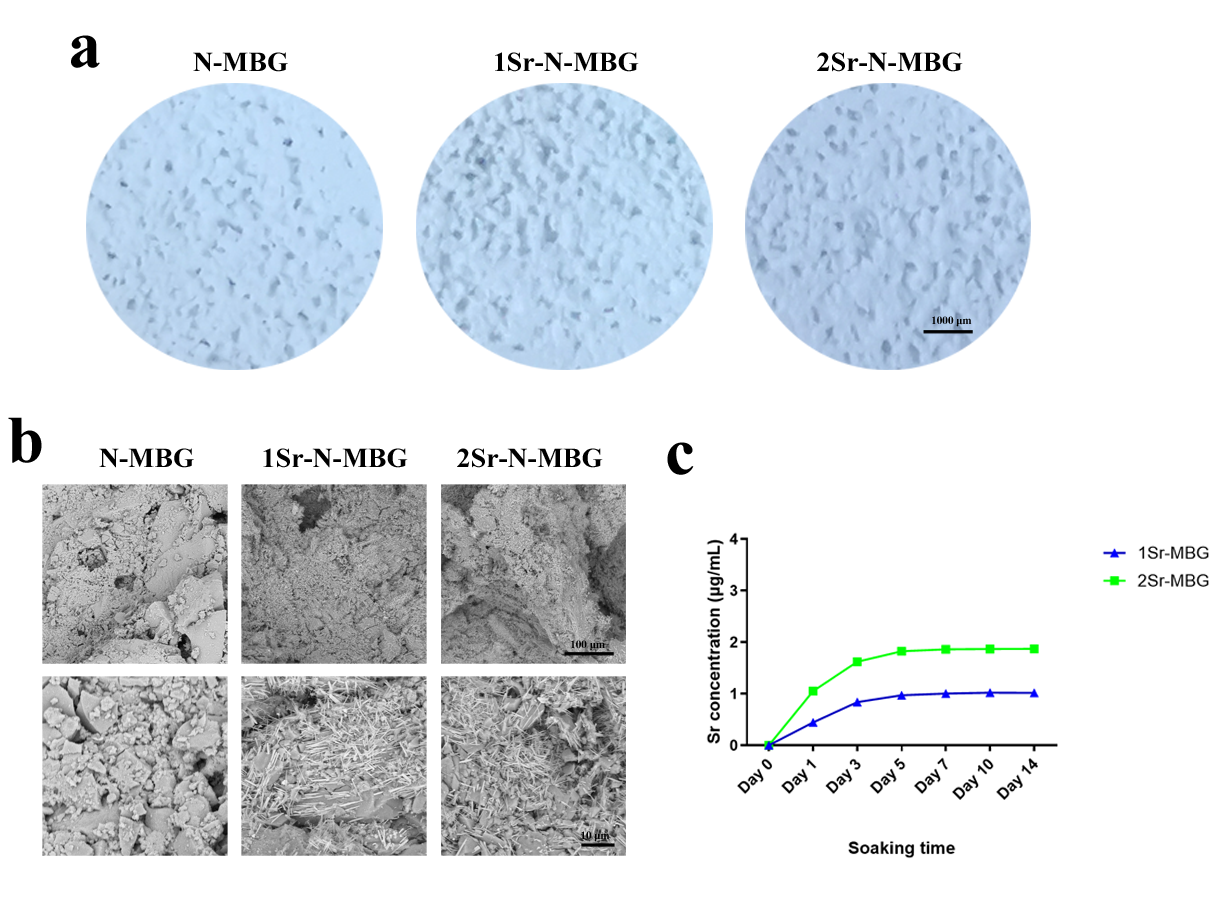


**Supplementary Fig. 1** **a** Representative images. **b** The morphology of each fabricated scaffold surface. **c** Sustained release pattern of Sr ion.


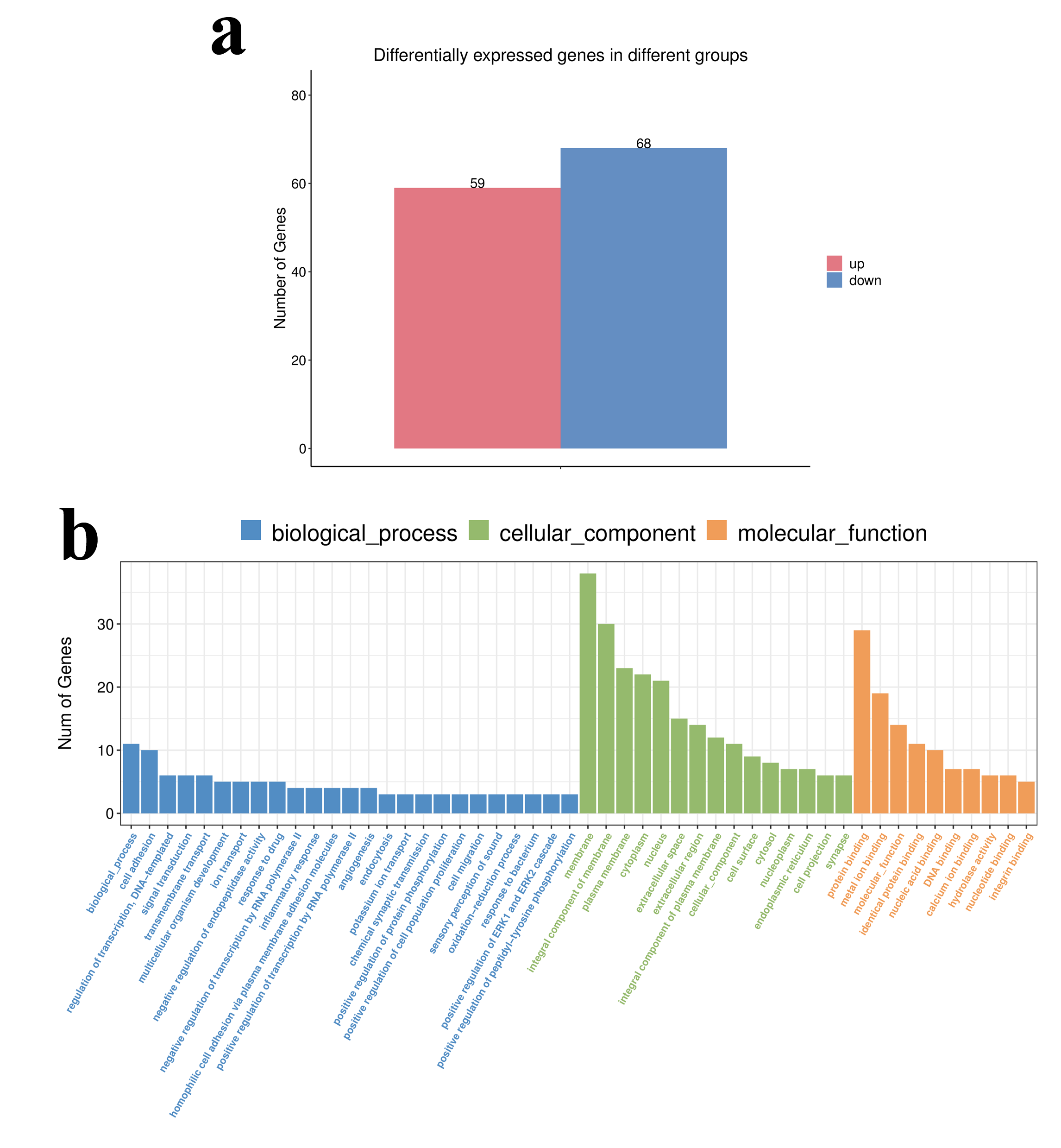


**Supplementary Fig. 2** RNA-seq analysis between N-MBG and 2Sr-N-MBG groups for comparison. **a** Differentially expressed genes. **b** GO enrichment analysis.
